# Insulin resistance, C-reactive protein, diastolic to systolic blood pressure ratio and epicardial fat are related to sedentary time, and inversely related to physical activity in school-aged children

**DOI:** 10.3389/fpubh.2024.1339860

**Published:** 2024-03-20

**Authors:** Fidanka Vasileva, Gemma Carreras-Badosa, Judit Bassols, Juan Serrano-Ferrer, Raquel Font-Lladó, Victor López-Ros, Inés Osiniri, Jose-Maria Martínez-Calcerrada, Marta San Millán, Abel López-Bermejo, Anna Prats-Puig

**Affiliations:** ^1^Pediatric Endocrinology Research Group, Girona Institute for Biomedical Research, Girona, Spain; ^2^University School of Health and Sport, University of Girona, Girona, Spain; ^3^Maternal-Fetal Metabolic Research Group, Girona Institute for Biomedical Research, Girona, Spain; ^4^Research Group of Culture and Education, Institute of Educational Research, University of Girona, Girona, Spain; ^5^Chair of Sport and Physical Education – Centre of Olympic Studies, University of Girona, Girona, Spain; ^6^Pediatrics, Clinica Bofill, Girona, Spain; ^7^Institute of Legal Medicine of Catalonia, Girona, Spain; ^8^Research Group of Clinical Anatomy, Embryology and Neuroscience, Department of Medical Sciences, University of Girona, Girona, Spain; ^9^Department of Medical Sciences, University of Girona, Girona, Spain; ^10^Pediatric Endocrinology, Dr. Josep Trueta Hospital, Girona, Spain

**Keywords:** epicardial fat, interventricular septal thickness, left ventricular posterior wall thickness, insulin resistance, C-reactive protein, physical activity, sedentary time, school-aged children

## Abstract

**Background:**

Physical activity (PA) is beneficial for the overall health. Objectives are: (1) To compare metabolic (MRM) and cardiovascular-risk-markers (CRM) in children according to their PA-level; (2) to explore the associations of MRM and CRM with PA and sedentary time (ST); and (3) to identify the associations between MRM and CRM in less (LA) and more active (MA) children.

**Methods:**

A total of 238 apparently healthy school-aged children were enrolled (132 boys/106 girls; 9.1 ± 1.8 years) and body mass index standard deviation score (BMI SDS) and blood pressure were assessed. Fasting venous blood sampling was performed to assess insulin resistance (HOMA-IR) and high-sensitivity-C-reactive protein (hsCRP). Epicardial fat, interventricular septal and left ventricular posterior wall thicknesses were assessed by high-resolution ultrasonography. PA and ST were assessed by enKid-questionnaire. Children were classified based on enKid-score as being LA and MA (below and above 50th percentile for PA).

**Results:**

MA-children had lower values for: BMI SDS, diastolic-to-systolic blood pressure ratio, HOMA-IR and hsCRP (7.02 to 61.5% lower, *p* = 0.040 to *p* < 0.0001) compared to LA-children. MRM and CRM were positively associated with ST (*p* = 0.003 to *p* < 0.001), and negatively associated with PA (*p* = 0.044 to *p* < 0.001). Finally, MRM were positively associated with CRM (*p* = 0.008 to *p* < 0.0001). Interestingly, the latter associations were observed in LA-children but were not present in MA-children.

**Conclusion:**

More PA is associated with better cardio-metabolic profile in school-aged children. PA seems to modulate the associations between MRM and CRM, thus reinforcing the idea that fostering PA in children may lower the risk for development of a cardio-metabolic disease.

## 1 Introduction

Physical activity (PA) is any movement produced by the skeletal muscles that raises energy expenditure above resting metabolic rate, and is performed to improve or maintain the components of physical fitness and health (1). Since PA is widely recognized as a primary contributor to overall health protection (2), the World Health Organization recommends ≥1 h/day of PA (3). In parallel, inactivity and sedentary time should be reduced because they promote obesity and diabetes (3). Sedentary time is characterized by any sitting, reclining, or lying posture with an energy expenditure ≤1.5 metabolic equivalents (4).

Nowadays, there is a growing prevalence of metabolic disorders in children that may place a child at a high risk of developing a cardiovascular disease (CVD) later in life. CVD are the most common cause of death globally, accounting for 17.8 million deaths worldwide (5). For instance, known metabolic risk markers such as insulin resistance (HOMA-IR) and high sensitivity C-reactive protein (hsCRP) are considered to induce detrimental effect on cardio-metabolic health (6). Increased HOMA-IR, together with increased total body adiposity and fat accumulation were related to increased epicardial fat (EF) in adult population (5, 7). Additionally, hsCRP was related to cardiac remodeling and diastolic dysfunction in hypertensive patients (8). Similar associations have been identified in children with insulin resistance, hypertension and CVD (9–11). However, to the best of our knowledge, no previous studies explored these associations in relation to PA in healthy children.

High blood pressure, especially diastolic-to-systolic blood pressure ratio (D/S BP ratio) has been proposed as a relevant cardiovascular risk marker because of its relation to systemic vascular resistance (12). EF, interventricular septal thickness (IVST) and left ventricular posterior wall thickness (LVPWT) are heart-related cardiovascular risk markers, and potential therapeutic targets for maintenance of cardiovascular health (13–15). Previous studies have shown that EF causes local inflammation and has direct effects on coronary atherosclerosis (13), while IVST and LVPWT predict all-cause death in patients with coronary artery disease (14), and cardiac events (15), respectively.

Since PA has been reported to be beneficial for the overall health (2), we hypothesize that there will be differences in the cardio-metabolic profile of the children according to their level of PA, and that more PA may relate to a better cardio-metabolic profile. Therefore, our objectives are: (1) To compare metabolic and cardiovascular risk markers in children according to the level of PA; (2) to explore the associations of metabolic and cardiovascular risk markers with PA and sedentary time; and (3) to identify the associations between metabolic and cardiovascular risk markers in less and more active children (below and above 50th percentile for PA).

## 2 Methods

### 2.1 Population and ethics

A total of 238 apparently healthy school-aged children (106 girls/132 boys; 9.19 ± 1.80 years) were recruited in primary health centers in Girona (Northeastern Spain). Inclusion criteria were: age between 5 and 12 years. Exclusion criteria were: (1) major congenital abnormalities; (2) chronic illness or chronic use of medication; (3) acute illness or use of medication in the last 2 weeks preceding potential enrolment; (4) abnormal blood counts, (5) abnormal liver, kidney or thyroid functions; and (6) hsCRP levels higher than 10 mg/L indicating an acute infection or inflammatory process (16). The research was approved by the Institutional Review Board of Dr. Josep Trueta Hospital, Girona, Spain (CEIC: 2010.056). Signed consent was obtained from the parents of all children included in the study.

### 2.2 Anthromopometric charactersitics and metabolic risk markers assessment

Body mass was measured wearing light clothes with a calibrated scale and height was measured with a Harpenden stadiometer. Body mass index (BMI) was calculated as body mass in kilograms divided by the square of height in meters. Age- and sex-adjusted standard deviation scores (SDS) for body mass, height and BMI were calculated using regional normative data (17). Venous blood sampling was performed in a fasting state in the morning (between 8.00 and 9.00 AM). Serum insulin was measured by immunochemiluminiscence (IMMULITE 2000, Diagnostic Products Corporation). Lower detection limit was 0.4 mIU/L and intra- and inter-assay coefficients of variability were less than 10%. Serum glucose was analysed by the hexokinase method (Cobas C; Roche Diagnostics, Indianapolis, US). Lower detection limit was 2.0 mg/dL and intra- and inter-assay coefficients of variability were less than 3%. HOMA-IR was estimated from fasting insulin and glucose concentrations using the homeostasis model assessment [HOMA-IR = (fasting insulin in mU/L) x (fasting glucose in mg/dL)/405]. Serum levels of hsCRP were measured using the ultrasensitive latex immunoassay CRP Vario (Sentinel Diagnostics, Abbott Diagnostics Europe, Milan, Italy). Lower detection limit was 0.2 mg/L and intra- and inter-assay coefficients of variability were less than 3%.

### 2.3 Cardiovascular risk markers assessment

Blood pressure was measured in a supine position on the right arm by means of an electronic oscillometer (Dinamap ProCare 100, GE Healthcare) with cuff size appropriate for the arm circumference. The average of three measurements was considered. Heart-related cardiovascular risk markers (EF, IVST and LVPWT) were measured using a high-resolution ultrasonography (MyLabTM25, Esaote, Firenze, Italy). To assess EF, IVST and LVPWT children were placed in the left lateral decubitus position according to the recommendations of the American Society of Echocardiography (18). Linear 7.5–12 MHz transducer was used for EF and a convex 3.5–5 MHz transducer was used for IVST and LVPWT. The measurements were taken from the parasternal long-axis views. Three consecutive measurements were performed and the calculated mean was considered for analysis. All measurements were taken on a separate visit and were performed by the same observer (a sonographer specialized in pediatric ecography) who was unaware of the clinical characteristics of the participants. Intra-observer coefficient of variation for ultrasound measurements was less than 6%.

### 2.4 PA and sedentary time assessment

PA and sedentary time were assessed by the enKid questionnaire that was filled out by the children’s parents (19). This self-reported questionnaire offers a broad, general perspective on children’s PA but it is practical, cost-effective and easy to apply, thus being convenient for PA assessment, especially in research including pediatric population (20). To analyze data, the following categories were created: (1) PA – hours per day spent playing alone, playing with others, and activities such as riding a bike, rollerblading, skateboarding, swimming, playing football, basketball, handball, volleyball etc. (2) sedentary time – hours per day in a seated position (screen time activities, listening to music, reading and studying).

### 2.5 Statistical analyses

Statistical analyses were performed using SPSS version 22.0 (SPSS Inc., Chicago, IL, United States). The normality of the data distribution was tested by the Kolmogorov–Smirnov test. Non-normally distributed variables were logarithmically transformed to improve the distribution symmetry. Subsequently, PA percentiles were created in order to classify children as more (above 50^th^ percentile) and less (below 50^th^ percentile) physically active. We employed this approach mainly because it provides balanced allocation by dividing the dataset into two equal parts, ensuring a reference threshold which is sample-specific, making it a suitable and convenient method for group formation in research (21). In this line, the use of percentiles is widely and commonly accepted in research with children, adolescents, adults and older population (21–28). Differences in metabolic and cardiovascular risk markers between more and less physically active children were examined with a Student’s *t*-test. It was followed by analysis of covariance to correct for potential confounding variables (age, sex and BMI). The associations of PA and sedentary time with metabolic and cardiovascular risk markers, as well as the associations between metabolic and cardiovascular risk markers, were analyzed by Pearson correlations. It was followed by a linear regression analysis (enter method) to correct for potential confounding variables (age, sex and BMI). Significance level was set at 0.05.

## 3 Results

Descriptive characteristics, as well as a comparison of the cardio-metabolic profile according to the level of PA of the children are presented in Table 1. Based on the results, more physically active children had lower values for BMI SDS (27.51% less, *p* = 0.040), insulin (30.72% less, *p* = 0.013), HOMA-IR (34.15% less, *p* < 0.001), hsCRP (61.54 less, *p* < 0.001) and D/S BP ratio (7.02% less, *p* < 0.0001) compared to less physically active children. Worthy to note is that we identified sex differences among the groups of more and less physically active children, thus we corrected the observed differences for any potential confounding variables such as age, sex and BMI. Interestingly, all differences remained significant in analysis of covariance correcting for the previously mentioned confounding variables (Table 1).

**Table 1:** Descriptive characteristics of the studied population and comparison of the cardio-metabolic profile between more and less physically active children.

Anthropometric characteristics and metabolic risk markers	All children (*N* = 238)	Less physically active children (below 50th percentile) (*N* = 119)	More physically active children (above 50th percentile) (*N* = 119)	Percentage of difference (%)	*p*-value
Age (years)	9.13 ± 1.79	9.25 ± 1.90	9.10 ± 1.66	1,63	0.516
Sex (M/F)	132/106	65/54	67/52	21.9	**0.040**
Body mass (kg)	44.7 ± 15.1	46.6 ± 15.8	44.0 ± 13.5	5.78	0.169
Body mass SDS	1.52 (0.45–2.59)	1.68 (0.58–2.62)	1.35 (0.40–2.53)	13.9	0.254
Height (cm)	139 ± 12	139.78 ± 12.51	139.55 ± 10.18	0.16	0.879
Height SDS	0.74 (0.04–1.57)	0.73 (0.08–1.51)	0.84 (−0.01–1.61)	9.88	0.566
BMI (kg/m2)	22.5 ± 4.8	23.1 ± 4.9	21.7 ± 4.0	6.52	0.016*
BMI SDS	1.49 (0.28–2.34)	1.73 (0.55–2.47)	1.42 (0.26–2.17)	27.5	0.040*
Insulin (ulU/mL)	9.14 ± 7.70	10.29 ± 8.79	7.55 ± 5.50	30.7	0.013*
Glucose (mg/dL)	87.8 ± 6.9	87.7 ± 6.8	88.0 ± 7.0	0.43	0.674
HOMA-IR	1.96 ± 1.53	2.16 ± 1.65	1.53 ± 1.08	34.2	<0.001*
HsCRP (mg/L)	1.50 (0.40–3.40)	1.75 (0.50–3.73)	0.95 (0.40–2.48)	61.5	<0.001*
Cardiovascular risk markers					
Systolic blood pressure (mmHg)	109.4 ± 11.5	110.2 ± 11.3	109.4 ± 10.8	0.76	0.569
Diastolic blood pressure (mmHg)	62.7 ± 8.2	64.6 ± 8.5	60.5 ± 7.4	6.51	<0.001*
D/S BP ratio (mmHg)	0.58 ± 0.07	0.59 ± 0.07	0.55 ± 0.06	7.02	<0.0001*
EF (cm)	0.29 ± 0.12	0.31 ± 0.12	0.29 ± 0.12	6.67	0.326
IVST (cm)	1.09 ± 0.41	1.08 ± 0.41	1.14 ± 0.40	5.41	0.287
LVPWT (cm)	1.26 ± 0.52	1.26 ± 0.53	1.32 ± 0.49	4.65	0.393
Physical (in)activity					
Sedentary time (hours/day)	10.4 ± 1.3	10.8 ± 1.3	9.8 ± 1.1	9.69	<0.0001*
PA (hours/day)	4.2 ± 1.0	3.8 ± 0.9	4.7 ± 0.9	19.9	<0.0001*

Data for Gaussian variables is presented as mean ± standard deviation. Data for non-Gaussian variables is presented as median and interquartile range. Significance level is set at 0.05 and significant values are marked in bold. *Significant when correcting for confounding variables: age, sex, BMI. BMI, body mass index; D/S BP ratio, diastolic-to-systolic blood pressure ratio; EF, epicardial fat; HOMA-IR, insulin resistance; HsCRP, high sensitivity C-reactive protein; IVST, interventricular septal thickness; LVPWT, left ventricular posterior wall thickness; PA, physical activity; SDS, standard deviation score.

**Figure 1:**
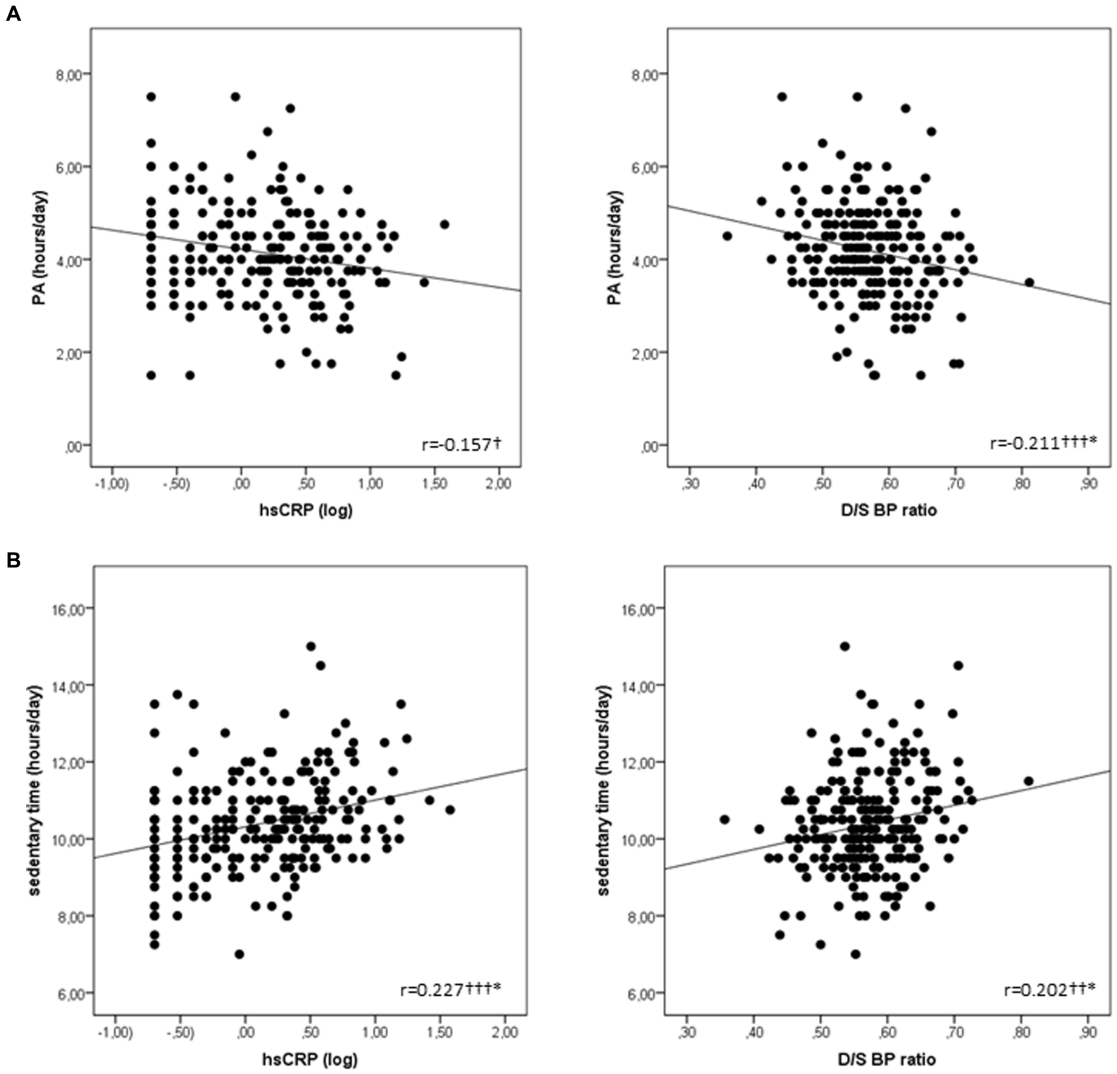
Scatterplots representing the associations of: **(A)** PA with hsCRP and D/S BP ratio; and **(B)** Sedentary time with hsCRP and D/S BP ratio. Significance level is set at 0.05 and significant values are marked as follows: *p* < 0.05†, *p* < 0.01†† and *p* < 0.001†††. *Associations that remained significant when correcting for confounding variables: age, sex and BMI. BMI, body mass index; D/S BP ratio, diastolic-to-systolic blood pressure ratio; hsCRP, high sensitivity C-reactive protein; PA, physical activity.

Bivariate associations of metabolic and cardiovascular risk markers with PA and sedentary time are presented in Table 2 and Figure 1. On the one hand, PA was negatively associated with HOMA-IR (*r* = −0.142, *p* = 0.044), EF (*r* = −0.133, *p* = 0.040) (Table 2), hsCRP (*r* = −0.157, *p* = 0.016) and D/S BP ratio (*r* = −0.211, *p* < 0.001) (Figure 1A). On the other hand, sedentary time was positively associated with insulin (*r* = 0.221, *p* < 0.001), HOMA-IR (*r* = 0.210, *p* = 0.003), EF (*r* = 0.197, *p* < 0.002) (Table 2), hsCRP (*r* = 0.227, *p* < 0.001) and D/S BP ratio (*r* = 0.202, *p* = 0.002) (Figure 1B). Worthy to note is that the negative association of D/S BP ratio with PA, as well as its positive association with sedentary time, remained significant after correcting for age, sex and BMI (Figure 1). The positive association between sedentary time and hsCRP also remained significant after correcting for age, sex and BMI (Figure 1B).

**Table 2:** Bivariate associations of metabolic and cardiovascular risk markers with PA and sedentary time in school-aged children (*N* = 238).

	PA (hours/day)		Sedentary time (hours/day)	
	*r*	*p*-value	*r*	*p*-value
Insulin log (ulU/mL)	−0.115	0.102	0.221	<0.001*
HOMA-IR	−0.142	**0.044**	0.210	**0.003**
EF (cm)	−0.133	**0.040**	0.197	**0.002**
IVST (cm)	−0.033	0.615	0.057	0.380
LVPWT (cm)	−0.002	0.979	0.045	0.494

Significance level is set at 0.05 and significant values are marked in bold. *Associations that remained significant when correcting for confounding variables: age, sex, BMI. BMI, body mass index; EF, epicardial fat; PA, physical activity; HOMA-IR, insulin resistance index; IVST, interventricular septal thickness; LVPWT, left ventricular posterior wall thickness.

**Figure 2:**
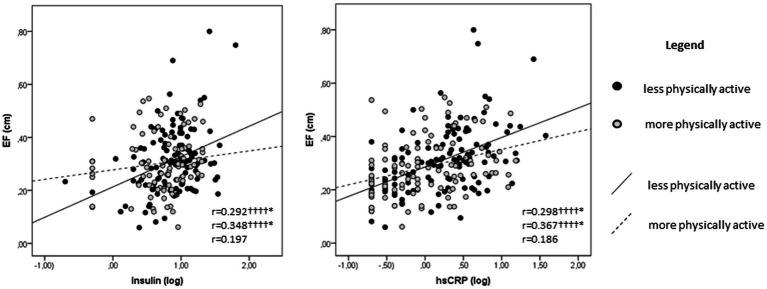
Scatterplots representing the associations of EF with insulin and hsCRP. Pearson’s correlation coefficients are presented in the following order from top to bottom: all studied children, less physically active children and more physically active children. Significance level is set at 0.05 and significant values are marked as follows: *p* < 0.0001††††. *Associations that remained significant when correcting for confounding variables: age, sex and BMI. BMI, body mass index; EF, epicardial fat; hsCRP, high sensitivity C-reactive protein.

Bivariate associations between metabolic and cardiovascular risk markers are presented in Table 3 and Figure 2. D/S BP ratio was positively associated with insulin (*r* = 0.196, *p* = 0.005) and HOMA-IR (*r* = 0.185, *p* = 0.008) in the studied children (Table 3). Furthermore, IVST and LVPWT were positively associated with hsCRP (*r* = 0.171, *p* = 0.008 and *r* = 0.186, *p* = 0.004) (Table 3), while EF was positively associated with insulin (*r* = 0.292, *p* < 0.0001) and hsCRP (*r* = 0.298, *p* < 0.0001) (Figure 2) in the same children. Interestingly, when we analyzed the same associations in groups according to the level of PA of the children, all previously mentioned associations were present only in less physically active children (*r* = 0.179 to *r* = 0.367, *p* = 0.049 to *p* < 0.0001), while they were not observed in more physically active children (Table 3 and Figure 2). Worthy to note is that the positive associations of EF with insulin and hsCRP remained significant after correcting for age, sex and BMI in the studied children, especially in less physically active children (Figure 2).

**Table 3:** Bivariate associations between metabolic and cardiovascular risk markers in apparently healthy school-aged children according to the level of PA.

	All children (*N* = 238)					
	D/S BP ratio (mm/Hg)		IVST (cm)		LVPWT (cm)	
	*r*	*p*-value	*r*	*p*-value	*r*	*p*-value
Insulin log (ulU/mL)	0.196	0.005*	0.001	0.998	0.004	0.960
HOMA-IR	0.185	**0.008**	−0.042	0.552	−0.033	0.642
HsCRP log (mg/L)	0.026	0.689	0.171	**0.008**	0.186	**0.004**

	Less physically active children (below 50th percentile) (*N* = 119)					
	D/S BP ratio (mm/Hg)		IVST (cm)		LVPWT (cm)	
	*r*	*p*-value	*r*	*p*-value	*r*	*p*-value
Insulin log (ulU/mL)	0.193	**0.034**	0.029	0.749	0.051	0.577
HOMA-IR	0.179	**0.049**	−0.037	0.690	0.004	0.964
HsCRP log (mg/L)	0.075	0.391	0.221	**0.010**	0.238	**0.006**

	More physically active children (above 50th percentile) (N = 119)					
	D/S BP ratio (mm/Hg)		IVST (cm)		LVPWT (cm)	
	*r*	*p*-value	*r*	*p*-value	*r*	*p*-value
Insulin log (ulU/mL)	0.064	0.568	0.035	0.756	−0.015	0.896
HOMA-IR	0.073	0.516	0.030	0.790	−0.026	0.815
HsCRP log (mg/L)	0.006	0.955	0.132	0.191	0.134	0.185

Significance level is set at 0.05 and significant values are marked in bold. *Associations that remained significant when correcting for confounding variables: age, sex, BMI. BMI, body mass index; D/S BP ratio, diastolic-to-systolic blood pressure ratio; HOMA-IR, insulin resistance index; HsCRP, high sensitivity C-reactive protein; IVST, interventricular septal thickness; LVPWT, left ventricular posterior wall thickness; PA, physical activity.

## 4 Discussion

Main findings of this study indicate that school-aged children who are more physically active have a better cardio-metabolic profile than those who are less physically active. Metabolic and cardiovascular risk markers are negatively related to PA and positively related to sedentary time in these children. Finally, metabolic risk markers were positively related to cardiovascular risk markers in the same children. Interestingly, PA seems to modulate the latter associations in a volume-dependent manner, as they were identified in less physically active children and were not present in more physically active children.

Children who are more physically active have lower insulin, HOMA-IR, hsCRP and D/S BP ratio, than less physically active children. This outcome allows us to accept the postulated hypothesis of the present study. Even if previous studies did not focus on comparing the metabolic and cardiovascular profile in healthy children depending on their PA levels, existing evidence in adult population is in line with our findings (29). For instance, previous studies reported that HOMA-IR, hsCRP and blood pressure were significantly lower in adults who perform a minimum of 150 min/week of PA, in comparison to those who performed less PA (29, 30). A recent meta-analysis confirmed that increased blood pressure in childhood is associated with higher risk for CVD and mortality in adulthood (31). However, subjects with increased blood pressure during childhood, but normal blood pressure during adolescence, were not at risk for developing a CVD (31). This evidence raises the need to develop an effective non-medication based strategy to reduce blood pressure in pre-pubertal children, with the aim to prevent CVD in adulthood. Results obtained in our study indicate that PA may be such strategy even in apparently healthy children, which emphasizes the importance for children to be physically active in order to maintain their cardiovascular health. As it has been previously suggested, PA may confer beneficial effects on metabolic and cardiovascular health by enhancing insulin sensitivity in the muscles and the liver (30). Moreover, PA helps reducing the amount of adipose tissue, which may prevent from obesity-driven insulin resistance and inflammation (30). In addition, our results reinforce the idea of benefits on cardiovascular health depending on the PA levels, which may be explained by a higher increase in the blood flow and a higher reduction in the vascular resistance due to the demand induced by higher PA levels (32).

Present findings also indicate that HOMA-IR, hsCRP, D/S BP ratio and EF are related to sedentary time in school-aged children. On the other hand, all these metabolic and cardiovascular risk markers were inversely related to PA. In line with the associations of metabolic and cardiovascular risk markers with PA and sedentary time in our study, previous evidence suggest that sedentary time is associated with altered metabolic profile (33), while PA was inversely related to BMI, fat percentage and HOMA-IR, thus lowering the risk for obesity in children (34). Additionally, higher levels of PA were related to lower hsCRP concentration (35). In the same line, cardiovascular markers were also negatively associated with PA. More precisely, the associations between D/S BP ratio and PA obtained in the present study are in accordance with previous reports suggesting that a lowered risk for hypertension was related to more PA and less sedentary time (36). In general, PA is associated with a lower heart rate on a long-term scale that could allow for sufficient time for diastolic left ventricular filling and coronary flow, therefore improving arterial compliance and lowering blood pressure (30, 37). Furthermore, it is worthy to emphasize that the World Hypertension League and the European Society of Hypertension recommended regular PA across the lifespan (including childhood) because of its well-established antihypertensive effects, as well as its favorable impact on other modifiable CVD risk factors (38). Moreover, they highlighted an inverse dose–response relationship between PA levels and CVD, i.e., the incidence of stroke, coronary artery disease and overall mortality (38). Finally, EF was negatively related to PA in our study suggesting that more PA will potentially contribute to less EF. To the best of our knowledge, no previous studies in healthy children have explored this association. However, in line with our findings, PA was related to lower visceral fat in Japanese female adults (39). One possible physiological mechanism explaining the inverse association between EF and PA may be the lipid mobilization triggered by increased catecholamine production during PA, leading to increased lipolysis rate in the EF cells (39).

Metabolic risk markers were related to cardiovascular risk markers in the present study, leading to the premise that any metabolic alterations in these children may potentially induce alterations in their cardiovascular health as well. Even though studies reporting associations between metabolic and cardiovascular risk markers in healthy children are scarce, previous studies have obtained similar results in adults (5, 7, 40), and in children at risk for developing a cardiovascular disease (9–11). For instance, increased body mass, insulin concentration and higher HOMA-IR in children with obesity were related to higher EF (9, 41). Increased hsCRP in hemodialysis patients was related to higher IVST (42), while higher BMI in children with hypertrophic cardiomyopathy was related to higher LVPWT (11). As discussed previously, studies in healthy children exploring the associations of metabolic risk markers with IVST and LVPWT, and in relation to PA, are missing to the best of our knowledge. However, we suggest that the associations between metabolic and cardiovascular risk markers identified in healthy children may be a result of the interplay between multiple underlying mechanisms. Higher insulin levels may lead to reduction of the sodium excretion rate and increased blood pressure (43). In addition, higher insulin levels may also promote fatty acid synthesis (44) which may end up in excessive lipid accumulation, therefore directly contributing to the formation of ectopic fat (45), such in this case - EF. Furthermore, hsCRP stimulates the production of adhesion molecules that enhance the infiltration of monocytes and lymphocytes resulting in fibrosis and cardiac remodeling (8), such as increased IVST and LVPWT. It is worthwhile to note that the results in our study were obtained in apparently healthy school-aged children, and thus indicate the importance of the early assessment of metabolic and cardiovascular profile in children.

Finally, it is also important to mention that the positive associations of D/S BP ratio with insulin and HOMA-IR, the positive associations of hsCRP with IVST and LVPWT, and the positive associations of EF with insulin and hsCRP identified in less physically active children, were not observed in more physically active children at the present study. More precisely, our results are indicative on a potential modulatory role of PA on the associations between the metabolic and cardiovascular risk markers because none of the associations identified in less physically active children was present in more physically active children. Moreover, current findings are pointing toward a dose-dependent modulatory effect of PA, propounding the idea that PA may modulate cardio-metabolic health in these children in a volume-dependent manner. Even though studies in healthy children that report volume-dependent PA-induced cardio-metabolic health benefits are scarce, in support to our findings previous studies in adult hypertensive population reported that higher PA levels provided superior cardio-metabolic benefits (38, 46). Additionally, the authors of the previous works argued that higher PA levels did not show any adverse effects among individuals diagnosed with hypertension, contradicting and disproving the hypothesis that there might be a threshold beyond which high PA levels could become harmful or counterproductive, especially for individuals with hypertension (38, 46). Remarkably, they demonstrated that higher PA levels are superior in reducing the risk for CVD and may serve as a beneficial approach in reducing the mortality rates among hypertensive population (38, 46). Indeed, prior findings in adult hypertensive population are in line with the findings of the present study in apparently healthy school-aged children. Therefore, taking in consideration previous and current findings, we suggest that encouraging higher levels of PA, even from pediatric age, could contribute to improved cardio-metabolic health outcomes in children, and potentially prevent the development of CVD in the future (38, 46).

## 5 Conclusion

More PA is associated with a better cardio-metabolic profile in school-aged children. PA appears to modulate the associations between metabolic and cardiovascular risk markers in a volume-dependent manner, thus reinforcing the idea that fostering PA in children and encouraging an active lifestyle from an early age, could potentially reduce the risk for future cardio-metabolic diseases and improve overall health outcomes in adulthood.

### 5.1 Practical applications

Present findings will potentially promote and inspire practicing more PA in order to evoke cardio-protective effects and maintain cardio-metabolic health in children. Also they may help the professional in exercise medicine, the coach and even the physical education teacher in the schools, to design appropriate PA interventions that will promote health and contribute to disease prevention in school-aged children. Also, these findings may raise the need for implementation of additional PA programs within schools that will result in higher PA levels, thus promoting a healthier lifestyle from a young age. Additionally, they may potentially encourage modifications in the physical education curriculum, advocating for the incorporation of more physical education classes, as well as more active breaks during the other classes with the aim to reduce the sedentary time.

Furthermore, current findings may raise awareness among parents about the importance of PA for children’s health and encourage them to guide and support their children to engage in PA beyond school hours. Community caregivers may be inspired to develop safe and accessible play areas in neighborhoods, parks, or public spaces to facilitate outdoor activities and playtime for children.

### 5.2 Limitations and future research

The major limitation of this study is the lack of accelerometer data in addition to the PA questionnaire in order to assess PA. Accelerometers may provide thorough assessment of PA, including information on PA intensity, offering a more detailed outlook than the self-reported questionnaires. However, the use of validated self-reporting instruments such as questionnaires is widely accepted in research as well as by medical communities, since they are practical, cost-effective and easy to apply, especially when dealing with large populations or specific demographics like children (20). Even though the self-reported questionnaires are commonly used in research due to their convenience, it is important to acknowledge their limitation to capture detailed information such as PA intensity, and point out the necessity for considering this in future research. Therefore, future studies should focus on clarifying the effects of light, moderate and vigorous PA on cardio-metabolic health. The impact of various PA intensities on cardio-metabolic health will potentially contribute in establishing intensity thresholds that are necessary to achieve prominent health benefits. This information is valuable for guiding public health recommendations and personalized exercise prescriptions. In addition, understanding the effects of different PA intensities on specific cardio-metabolic health indicators, may contribute to the development of personalized exercise programs for individuals with specific health conditions. Finally, the acquired knowledge can be translated into tailored recommendations, strategies and exercise interventions with the aim to improve public health and reduce the burden of cardio-metabolic diseases.

Furthermore, future interventional studies should also compare the effects of different types of physical exercise (e.g., aerobic, anaerobic, mixed interventions) on cardio-metabolic health. These studies could clarify further the physiological responses to varying exercise regimens. This may contribute significantly in optimizing exercise prescriptions designed to maintain and improve cardio-metabolic-health. In addition, the acquired knowledge may raise the need for further studies that will implement specifically tailored PA interventions in school settings, with the aim to assess their impact on improving cardio-metabolic health in children.

Another limitation of the present study is the cross-sectional design which does not allow inference on causality to be done. Alternatively, longitudinal follow-up studies track participants over time and may offer insights into the causality of sustained PA and the long-term cardio-metabolic health benefits. Thus, further longitudinal studies should be designed to observe changes in PA patterns, and elucidate the underlying mechanisms for the long-term benefits that may be induced by PA.

Future experimental and genome-wide studies should investigate the epigenetic modifications potentially induced by PA in children, and how these modifications may impact their cardio-metabolic health. By pursuing these research avenues, researchers can uncover the epigenetic mechanisms underlying the relationship between PA and cardio-metabolic health, and potentially develop interventions that will evoke the desired modifications to maximize cardio-metabolic health outcomes.

Finally, further comparative studies should aim to explore potential sex-specific differences in PA-induced cardio-metabolic health benefits. Future research employing larger sample size should focus on comparing the effects of PA on cardio-metabolic health in males and females separately in order to understand potential sex-specific implications.

## Data availability statement

The original contributions presented in the study are included in the article/supplementary material, further inquiries can be directed to the corresponding author.

## Ethics statement

The studies involving humans were approved by Institutional Review Board of Dr. Josep Trueta Hospital, Girona, Spain. The studies were conducted in accordance with the local legislation and institutional requirements. Written informed consent for participation in this study was provided by the participants’ legal guardians/next of kin.

## Author contributions

FV: Conceptualization, Formal analysis, Investigation, Methodology, Software, Writing – original draft. GC-B: Conceptualization, Data curation, Formal analysis, Investigation, Methodology, Software, Writing – review & editing. JB: Data curation, Funding acquisition, Project administration, Writing – review & editing. JS-F: Formal analysis, Methodology, Software, Writing – review & editing. RF-L: Methodology, Software, Supervision, Writing – review & editing. VL-R: Methodology, Software, Writing – review & editing. IO: Data curation, Methodology, Software, Writing – review & editing. JM-C: Data curation, Methodology, Software, Writing – review & editing. MS: Methodology, Writing – review & editing. AL-B: Conceptualization, Data curation, Formal analysis, Funding acquisition, Investigation, Methodology, Project administration, Supervision, Writing – review & editing. AP-P: Conceptualization, Data curation, Formal analysis, Investigation, Methodology, Software, Supervision, Writing – review & editing.
